# T215. CLINICAL PREDICTORS OF HOSPITALIZTIONS IN FIRST EPISODE PSYCHOTIC PATIENTS: A NATURALISTIC FOLLOW UP STUDY

**DOI:** 10.1093/schbul/sby016.491

**Published:** 2018-04-01

**Authors:** Olga Sparano, Pau Soldevila-Matias, Carlos Gonzalez, Lucia Bonet, Esther Lorente, Jose Miguel Carot, Julio Sanjuan

**Affiliations:** 1 Hospital Universitario de la Ribera; 2 Hospital Clinico INCLIVA; 3 Universidad Valencia; 4 Hospital Clinico INCLIVA, CIBERSAM; 5 Universidad Politecnica

## Abstract

**Background:**

Some naturalistic longitudinal studies of first psychotic episodes of the last 50 years have suggested associations between psychopathology and the remission of symptoms and the clinical course of disease.1 A recent study in a large sample of patients with schizophrenia has obtained significant results using the number of hospitalizations as outcome variable.2

The main objective of this study is to know if clinical and sociodemographic variables predict the number of hospitalizations after the first psychotic episode

**Methods:**

Naturalistic, longitudinal follow-up study in a sample of 212 patients of first-episode psychosis attending public mental health service in Area 5 of Valencia (Spain) in a period between 2010–2017. Of 212 patients, a total of135 were included, excluding patients lost due to abandonment and death.

The study included a) baseline variables: sociodemographic, risk factors (Cannnabis use), clinical scales; PANSS, CGI (clinical global impression) and GAF (global assessment of functioning scale) and kind of treatment (oral versus injectable). b) outcome variables: number of visits to the emergency room, hospitalizations, and outpatient consultations.

**Results:**

None of the psychopathological or treatment variables at baseline were significantly associated with the outcome variables.

The younger patients have a significant (p < 0.01) higher number of emergencies room visit in the follow up.

**Discussion:**

In contrast with previous reports1,2 Tihonen J et al2017)) we were not able to find any relationship between severity of illness (at baseline) or the kind of treatment (oral versus injectable) with the emergency rooms visits or number of hospitalizations.

The only significant result was related with the age of the patients. Younger patients have more probability of having more visit to the emergency room.

**References:**

1. Capdeville D. A multi-dimensional approach to insight and its evolution in first- episode psychosis: a 1 -year outcome naturalistic study. Psychiatry Res. 2013 dec 30;210(3):835–41

2. Tihonen J.Real-World effectiveness of antipsychotic treatments in a Nation wide Cohort of 29.823 patients with schizophrenia. Jamapsych.2017;74(7)686–693

